# Supplementary material for: Perception and Educational Needs of Developmentally Supportive Care At-Home for Parents of Pre-Term Newborns
Source: Healthcare (Basel). 2023 Jun 9;11(12):1700. doi: 10.3390/healthcare11121700 (PMC10298373; doi:10.3390/healthcare11121700)
Supplement: Supplementary file 1 [file healthcare-11-01700-s001.zip › healthcare-2403048-supplementary.pdf]

Question1. "Do you know about developmentally supportive care at home?"

- Do you know about neurologic developmental care at home?
- Do you know about feeding and nutritional care at home?
- Do you know about medical management care at home?

Question2. "What are the greatest challenges you face as you parent your child?"

- What are the greatest challenges you face as you about your child?
- What are the greatest challenges you face as you about your home environment?

Question3. "What contents do you wish to be included in the education about developmentally supportive care?"

- What would you like to know about rehabilitation section?
- What would you like to know about emotional support section?
- What would you like to know about social support section?
